# Development and Validation of Ischemic Events Related Signature After Carotid Endarterectomy

**DOI:** 10.3389/fcell.2022.794608

**Published:** 2022-03-17

**Authors:** Chunguang Guo, Zaoqu Liu, Can Cao, Youyang Zheng, Taoyuan Lu, Yin Yu, Libo Wang, Long Liu, Shirui Liu, Zhaohui Hua, Xinwei Han, Zhen Li

**Affiliations:** ^1^ Department of Endovascular Surgery, The First Affiliated Hospital of Zhengzhou University, Zhengzhou, China; ^2^ Department of Interventional Radiology, The First Affiliated Hospital of Zhengzhou University, Zhengzhou, China; ^3^ Institute for Cardiovascular Physiology, Goethe University, Frankfurt, Germany; ^4^ Department of Cardiology, The First Affiliated Hospital of Zhengzhou University, Zhengzhou, China; ^5^ Department of Cerebrovascular Disease, Zhengzhou University People’s Hospital, Zhengzhou, China; ^6^ Department of Pathophysiology, School of Basic Medical Sciences, The Academy of Medical Science, Zhengzhou University, Zhengzhou, China; ^7^ Department of Hepatobiliary and Pancreatic Surgery, The First Affiliated Hospital of Zhengzhou University, Zhengzhou, China

**Keywords:** ischemic events, carotid endarterectomy, diagnosis model, machine leaning, immune infiltration

## Abstract

**Background:** Ischemic events after carotid endarterectomy (CEA) in carotid artery stenosis patients are unforeseeable and alarming. Therefore, we aimed to establish a novel model to prevent recurrent ischemic events after CEA.

**Methods:** Ninety-eight peripheral blood mononuclear cell samples were collected from carotid artery stenosis patients. Based on weighted gene co-expression network analysis, we performed whole transcriptome correlation analysis and extracted the key module related to ischemic events. The biological functions of the 292 genes in the key module were annotated via GO and KEGG enrichment analysis, and the protein-protein interaction (PPI) network was constructed via the STRING database and Cytoscape software. The enrolled samples were divided into train (*n* = 66), validation (*n* = 28), and total sets (*n* = 94). In the train set, the random forest algorithm was used to identify critical genes for predicting ischemic events after CEA, and further dimension reduction was performed by LASSO logistic regression. A diagnosis model was established in the train set and verified in the validation and total sets. Furthermore, fifty peripheral venous blood samples from patients with carotid stenosis in our hospital were used as an independent cohort to validation the model by RT-qPCR. Meanwhile, GSEA, ssGSEA, CIBERSORT, and MCP-counter were used to enrichment analysis in high- and low-risk groups, which were divided by the median risk score.

**Results:** We established an eight-gene model consisting of *PLSCR1*, *ECRP*, *CASP5*, *SPTSSA*, *MSRB1*, *BCL6*, *FBP1*, and *LST1*. The ROC-AUCs and PR-AUCs of the train, validation, total, and independent cohort were 0.891 and 0.725, 0.826 and 0.364, 0.869 and 0.654, 0.792 and 0.372, respectively. GSEA, ssGSEA, CIBERSORT, and MCP-counter analyses further revealed that high-risk patients presented enhanced immune signatures, which indicated that immunotherapy may improve clinical outcomes in these patients.

**Conclusion:** An eight-gene model with high accuracy for predicting ischemic events after CEA was constructed. This model might be a promising tool to facilitate the clinical management and postoperative surveillance of carotid artery stenosis patients.

## 1 Introduction

Ischemic events, mainly ischemic heart disease and ischemic stroke, are the leading cause of death and disability worldwide (Murray and Lopez, 1997; Campbell et al., 2019). The main etiology of ischemic events is atherosclerosis formation, which arises from inflammation, lipid deposition and plaque fibrosis in the vascular endothelium over decades (Franceschini et al., 2018; Libby et al., 2019). Therefore, it is necessary to accurately identify atherosclerotic patients who are more prone to ischemic events. With developments in medicine and technology, multiple diagnostic techniques have been used to identify people at high risk of ischemic events, including noninvasive (such as computed tomography, biomarkers, stress testing and nuclear scanning) and invasive (such as selective and superselective arteriography) techniques (Dagvasumberel et al., 2012; Zhang et al., 2017; Martinez et al., 2020; Varasteh et al., 2021). Nonetheless, these methods have only moderate prediction accuracies, and some high-risk patients are not identified early, which leads to ischemic events (Penalvo et al., 2016). Thus, new methods to identify patients at high risk for ischemic events are urgently needed.

Indeed, substantial efforts have been made to cope with the occurrence of ischemic events, and carotid endarterectomy (CEA) has been indicated to be one of the most critical techniques (Howell, 2007). Unstable plaque shedding of the carotid intima is an important cause of cardiovascular and cerebrovascular occlusion, especially at the carotid bifurcation. In clinical practice, CEA is the optimal treatment modality to prevent ischemic events in patients with atheromatous disease at the carotid bifurcation (Howell, 2007; Rerkasem et al., 2020). However, this prophylactic surgery does not provide complete prevention, and some patients who undergo regular CEA surgery may still experience ischemic events (Folkersen et al., 2012). The recurrence of ischemic events (including ischemic stroke and myocardial infarction) after CEA is an important and urgent issue, but few studies have focused on it (Folkersen et al., 2012; Zenonos et al., 2012).

With the development of bioinformatics and machine learning, elegant studies have broadly applied artificial intelligence in the medical field because it better describes the complexity and unpredictability of human physiology (Deo, 2015; Rajkomar et al., 2019). Compared with traditional imaging diagnostic methods, machine learning can extract the most critical characteristics of the disease from high-dimensional variables to improve the performance of predicting ischemic events after CEA (Heo et al., 2019). With the help of these algorithms (such as random forest (Svetnik et al., 2003), and least absolute shrinkage and selection operator (LASSO) (Tibshirani, 1997)), researchers could identify the key factors in predicting recurrent ischemic events after CEA from massive data.

In this study, we retrieved gene expression data and clinical information from GEO for 97 patients. The hub genes of recurrent ischemic events after CEA were identified by machine learning algorithms and validated in an independent cohort (which included 50 samples from our hospital). Finally, we developed and validated a diagnostic model for predicting the probability of ischemic events after CEA. Based on this model, it is possible to intervene recurrent ischemic events after CEA in advance and improve clinical outcomes.

## 2 Materials and Methods

### 2.1 Data Source

**FIGURE 1:**
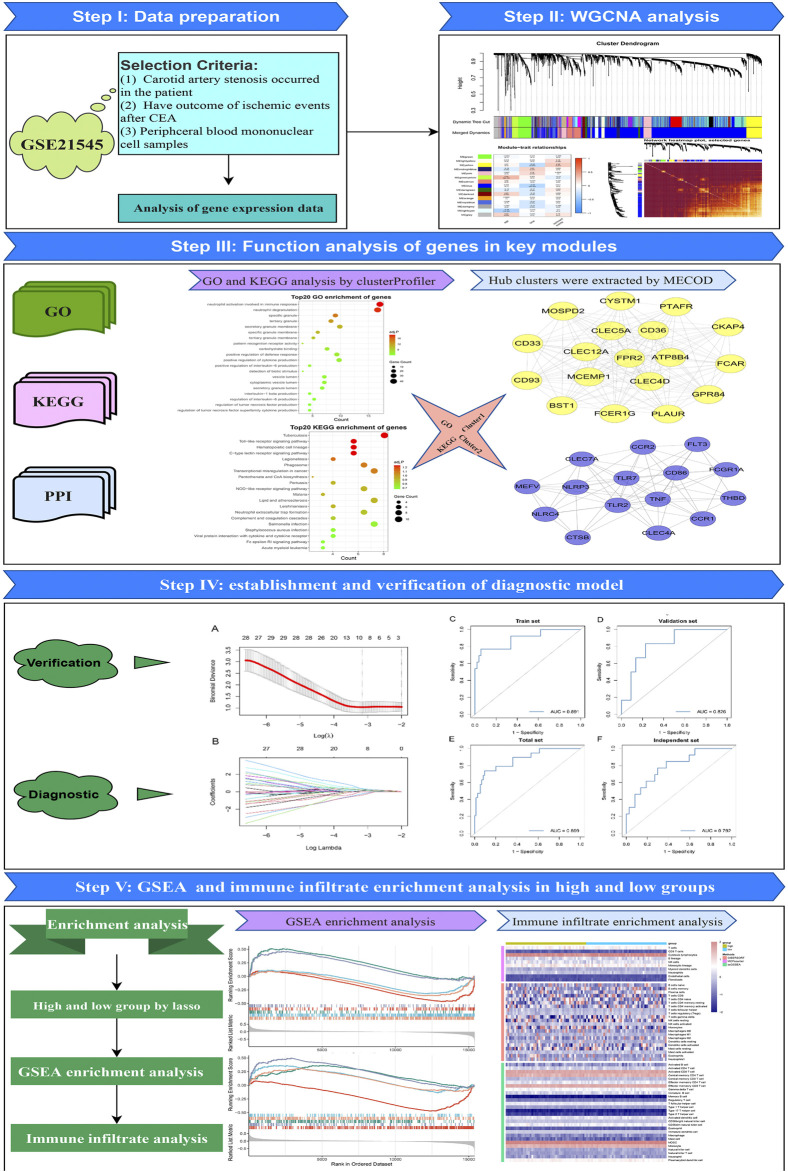
Flowchart of the analysis procedure.

The workflow of the overall analysis is shown in Figure 1. The gene expression and clinical annotation data of GSE21545 (Folkersen et al., 2012) were retrieved from the Gene Expression Omnibus (GEO) database. This dataset was based on an Affymetrix^®^ platform (GPL570) and included 97 peripheral blood mononuclear cell samples. The raw data were processed using the robust multichip analysis (RMA) algorithm implemented in the “Affy” R package. RMA was used to perform background adjustment, quantile normalization, and final summarization of oligonucleotides per transcript using the median polish algorithm (Liu et al., 2022a). The baseline clinical data of patients were presented in Supplementary Table S1.

### 2.2 Weighted Gene Co-Expression Network Analysis

Based on gene expression profiles, a total of 22,880 genes were identified from the samples. All genes were sorted in descending order according to their expression variability, which was calculated by the median absolute deviation in the entire dataset. To ensure the rationality of network construction, we excluded the outlier samples using an optimal version of hierarchical clustering, which applied Euclidean distance and averaging methods to rearrange the samples. Next, based on the top 5,000 genes, a gene co-expression network was constructed using the “WGCNA” R package (Langfelder and Horvath, 2008). We used step-by-step methods to construct gene networks. To meet the criterion of scale-free network distribution, the Pearson correlation coefficient between paired genes was calculated, and the optimum soft threshold *β* was selected. First, the Pearson’s correlation value between paired genes was used to acquire a similarity matrix. Next, with the optimum soft threshold value, the similarity matrix was transformed to an adjacency matrix. The adjacency matrix was calculated by setting the parameter amn = |cmn| *β* (cmn = Pearson’s correlation between genes m and n; amn = adjacency between genes m and n). Subsequently, the adjacency matrix was transformed into a topological overlap matrix (TOM), which was used to describe the similarity of gene expression, and 1-TOM was used to describe the dissimilarity between genes. Finally, a dynamic tree algorithm was used to partition the modules of the hierarchical clustering results (minimum module size = 30; deep-split = 2; cut tree height = 0.99; merge module height = 0.25). To further investigate the module, the dissimilarity of the module eigengene (ME) was calculated. A cut line for the module dendrogram was selected, and then the modules with cutting height <0.25 were merged (Guo et al., 2022).

### 2.3 Identification of Clinically Significant Modules

MEs were used for the component analysis of each module, and modules with similar expression profiles showed highly correlated eigengenes. The relevant modules were identified by calculating the correlation between the ME and ischemic events. The gene module with the highest correlation coefficient and a *p* < 0.05 was considered the most relevant module to ischemic events and was defined as the key module.

### 2.4 Protein-Protein Interaction Network Construction

All genes in the key module with a minimum level of confidence greater than 0.4 were submitted to the Search Tool for the Retrieval of Interacting Genes/Proteins (STRING) (https://string-db.org/) database version 11.0 (Szklarczyk et al., 2017; Szklarczyk et al., 2019). Protein interaction data obtained from the STRING database were used to calculate the degrees of genes by Cytoscape software (version 3.8.0; https://cytoscape.org/) (Shannon et al., 2003). Based on the maximal clique centrality (MCC) algorithm, significant modules with strong protein interactions were calculated and selected by Molecular Complex Detection (MCODE), which is a plugin in Cytoscape. The parameter settings for MCODE were as follows: degree cut ≥2, K-core ≥2, node score cut ≥2, and maximum depth = 100.

### 2.5 Functional Enrichment Analysis

“ClusterProfiler” (Yu et al., 2012; Liu et al., 2021a), a Bioconductor package, was used to perform Gene Ontology (GO) and Kyoto Encyclopedia of Genes and Genomes (KEGG) pathway enrichment analysis. The terms with *p <* 0.05 were considered significant.

#### 2.6 Random Forest

To identify genes associated with ischemic events, random forest was employed. Random forest, originally proposed by Breiman (Mantero and Ishwaran, 2021), is an ensemble learning algorithm that can construct abundant trees and predict outcomes by voting across all trees. In this study, the expression values of all genes in the key module were extracted and merged with the clinical characteristic information of the samples. Then, all samples were randomly divided into train (75% of samples, n = 66) and validation datasets (25% of samples, *n* = 28). Finally, “randomForestSRC” version 2.9.3 (which provides fast computing of unified random forests for survival, regression, and classification), a package in R, was used to screen out key genes associated with ischemic events in the train dataset.

### 2.7 LASSO Logistic Regression Model

To further identify genes associated with ischemic events after CEA, the LASSO regression algorithm (Lockhart et al., 2014) was used to obtain the coefficient for each key gene selected by random forest. To achieve this purpose, we used the “glmnet” (Friedman et al., 2010) R package (which is used for LASSO and elastic-net regularized generalized linear models). The alpha parameter of glmnet was set to 1, and the lambda value was chosen by cross-fold validation of the key gene set (5-fold cross-validation). Ultimately, the diagnostic model achieved the best lambda value, and its predictive accuracy in the train and validation sets was assessed by receiver operating characteristic (ROC) curve and precision recall (PR) curve.

### 2.8 Human Carotid Artery Stenosis Specimens

Participants fulfilling all of the following inclusion criteria are eligible for the study: 1) Imaging revealed carotid artery stenosis; 2) have clearly defined indications for surgery; 3) Patients with valvular heart disease, blood diseases, and malignant tumors were excluded. A total of 50 peripheral venous blood samples were collected from patients with carotid stenosis in the First Affiliated Hospital of Zhengzhou University. The baseline clinical data of patients were presented in Supplementary Table S1. The specimens obtained upon admission to the hospital and stored at −80°C until use in quantitative real-time qPCR (RT-qPCR). The Research Ethics Committee of the First Affiliated Hospital of Zhengzhou University approved this study, which was consistent with the Declaration of Helsinki, and the TRN is 2019-KW-94.

### 2.9 RNA Isolation and RT-qPCR

Total RNA was isolated from peripheral blood using RNAiso Plus (Takara, Dalian, China) according to the manufacturer’s instructions. The integrity and purity of the extracted total RNA were measured using NanoDrop One (Thermo Fisher Scientific, Waltham, United States) ultra-micro UV spectrophotometer. Reverse transcription was performed using the PrimeScript RT reagent Kit (Takara, Dalian, China) with gDNA Eraser. Serum RNA was reverse transcribed into cDNA using a RevertAid H Minus First Strand cDNA Synthesis Kit (Thermo Fisher Scientific, Waltham, United States) under the following conditions: 25°C for 5 min, 42°C for 60 min, and 70°C for 5 min. The product was immediately stored at −80°C until use.

The RT-qPCR was performed on a QuantStudio five Real-Time PCR System (Applied Biosystems, Foster City, United States) using a Hieff qPCR SYBR Green Master Mix kit (Yeasen, Shanghai, China). The RT-qPCR reaction was performed 95°C for 5 min, followed by 40 cycles of 95°C for 10 s and a primer-specific annealing temperature of 60°C for 30 s. The RT-qPCR primer sequences were provided in Supplementary Table S1. The relative quantification values for RNA were calculated by the 2-ΔΔCt method. GAPDH was used as an endogenous control for normalization.

### 2.10 Gene Set Enrichment Analysis and Immune Infiltration Profiles

Based on the median risk score of each sample, the entire study cohort was divided into high- and low-risk groups. The differential genes between the high- and low-risk groups were identified by the “limma” package and sequenced by the log2 (fold change) value. GSEA was used to decipher the underlying biological mechanisms of the genes in this model using GO and KEGG terms (Molecular Signatures database, version: c5. go.v7.4. symbols.gmt and c2. cp.kegg.v7.4. symbols.gmt). After that, the CIBERSORT (Newman et al., 2015; Liu et al., 2022b), MCP-counter (Shi et al., 2020; Liu et al., 2021b) and single-sample gene set enrichment analysis (ssGSEA) (Yi et al., 2020; Liu et al., 2022c) algorithms were used to explore the infiltration abundance of different immune cells between the high- and low-risk groups. Heatmaps and boxplots were used to uncover the degree of difference in the responses of various immune cell subsets between the two groups under different algorithms.

### 2.11 Statistical Analysis

All data processing, statistical analyses and plotting were completed using the R program (version 4.03). The unpaired Student’s t-test and Wilcoxon test were used to compare the differences between two groups. The Benjamin-Hochberg method was used to further calculate the false discovery rate (FDR). For every analysis, statistical significance was considered at *p* < 0.05.

## 3 Results

### 3.1 Preparation of Data for WGCNA

In this section, we cleaned the raw gene profiles for WGCNA. Based on the 97 PBMC samples with 22,880 gene expression profiles, we calculated the median absolute deviation (MAD) of each gene and retained the top 5,000 genes sorted by the MAD. The hierarchical clustering algorithm was further used for three outlier samples. After removing the three samples, we obtained a clean dataset consisting of 94 PBMC samples with 5,000 gene expression profiles.

### 3.2 Co-Expression Network Construction

**FIGURE 2:**
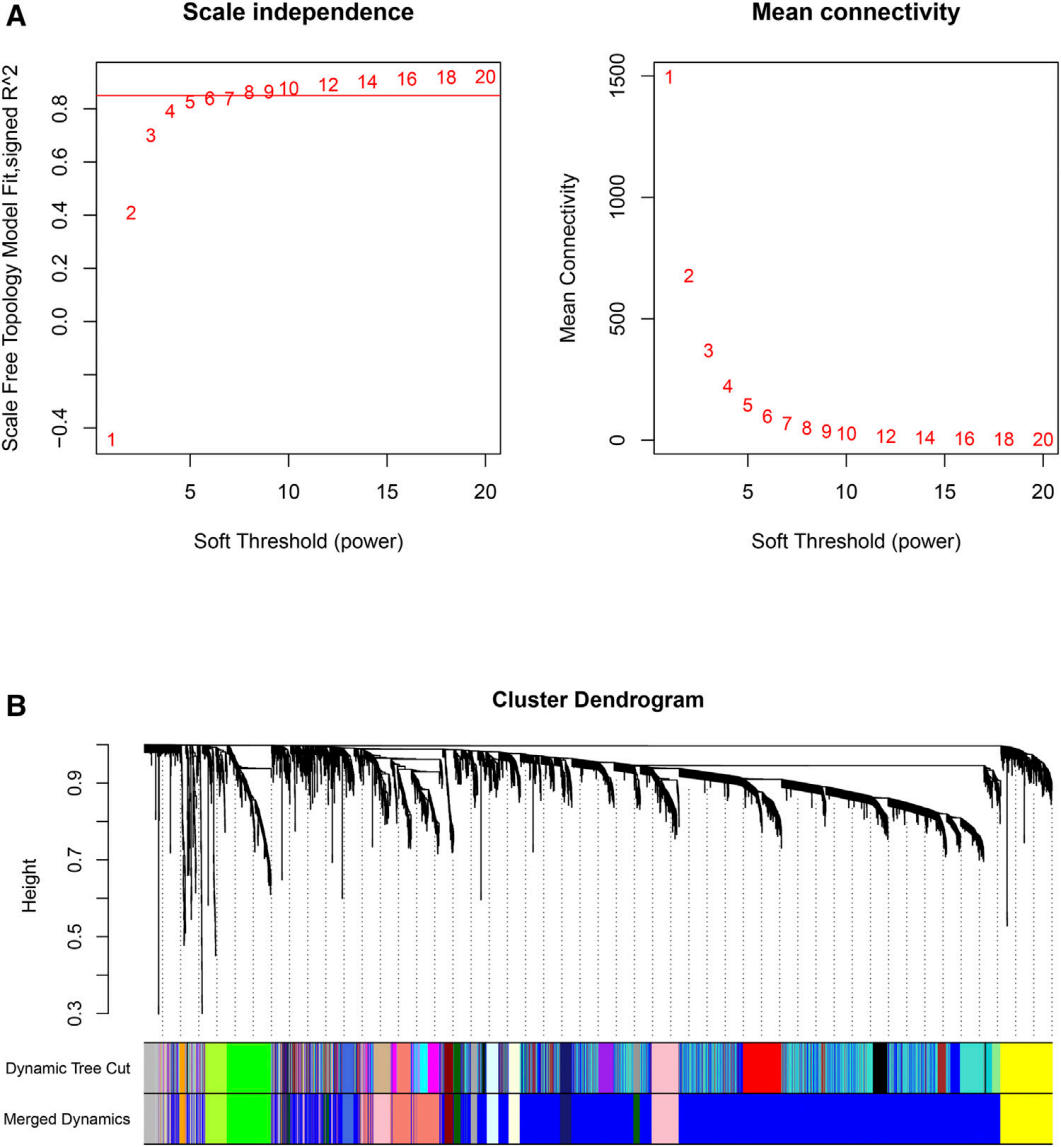
Scale-free networks were constructed, and genes were clustered by WGCNA. **(A)**: Scale-free network analysis under different soft-thresholding powers. The left panel shows scale-free topological indices at different soft-thresholding powers. The right panel shows the correlation analysis between the soft-thresholding powers and average connectivity of the network. **(B)**: Gene clustering diagram based on hierarchical clustering under optimal soft-thresholding power. (Dynamic Tree Cut: before module merging; Merged Dynamics: after module merging).

**FIGURE 3:**
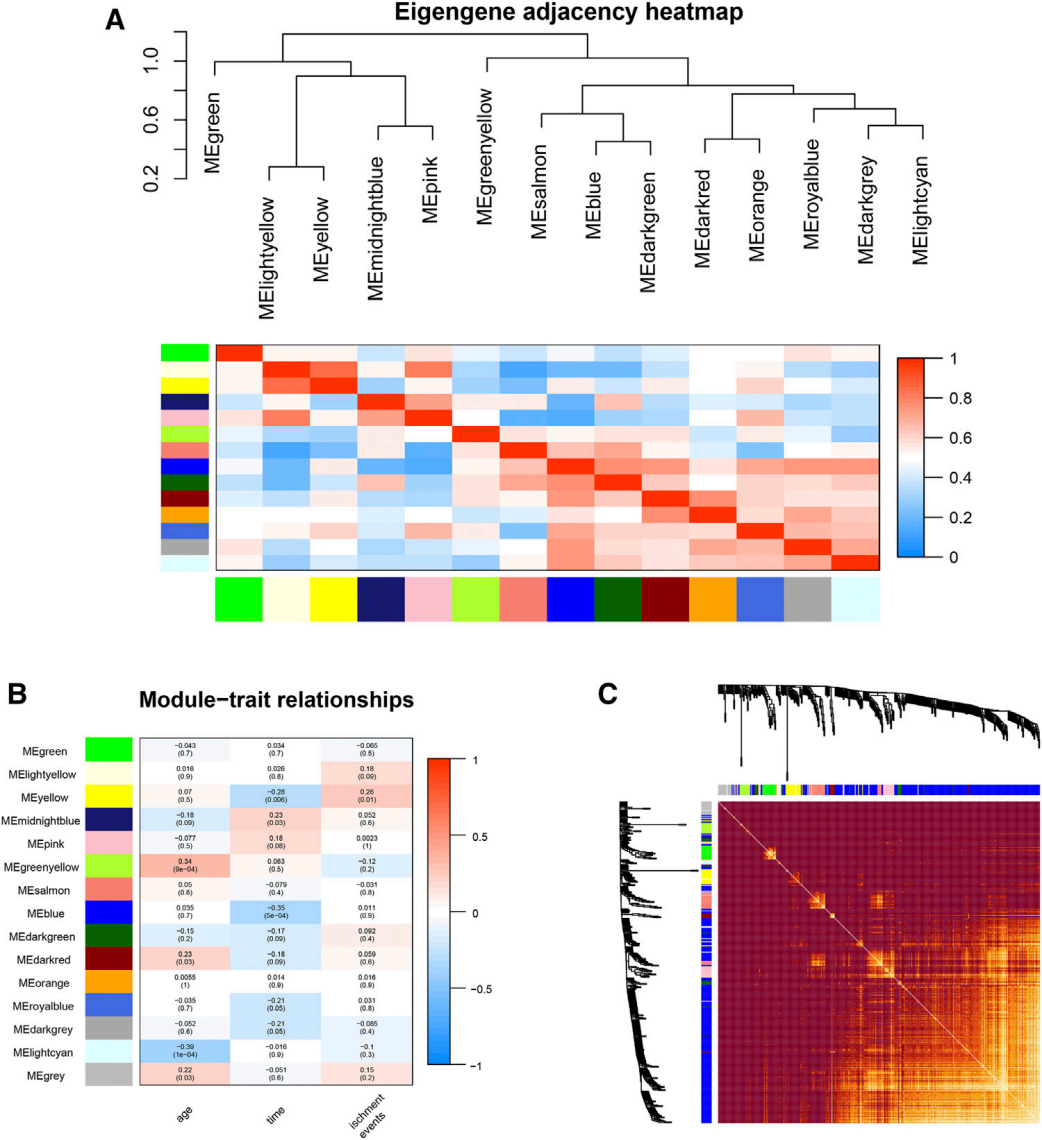
Gene module analysis based on WGCNA. **(A)**: Heat map of the eigengene adjacency. **(B)**: Heatmap between gene modules and clinical characteristics. **(C)**: Heatmap of the topological overlap matrix of genes selected by WGCNA.

First, the pickSoftThreshold function (from the “WGCNA” R package) was used to select the optimal soft threshold. Under the premise that the absolute value of the correlation coefficient is greater than 0.8, we chose 8 as the optimal soft threshold for constructing scale-free networks (Figure 2A). Next, we employed the cutreeDynamic function (from the “dynamicTreeCut” R package) to identify co-expression modules in the network (Figure 2B), and all genes were clustered among the 26 modules. To reduce the complexity of the network, modules with similarity greater than 0.75 were merged. MergeCloseModules, a function in the “WGCNA” R package, was used to merge these modules (cutHeight = 0.25, verbose = 3), and only 15 modules remained. The number of genes in each module is displayed in Table 1. After merging the modules, cluster dendrograms were plotted by the plotDendroAndColors function (from the “WGCNA” R package) (Figure 2B). Ultimately, the heatmap depicted the TOM among 400 genes (which were randomly selected from all genes) in WGCNA (Figure 3C).

**TABLE 1:** Number of genes contained in the merged module.

Modules	Numbers	Modules	Numbers	Modules	Numbers
Blue	2,981	Grey	176	Lightyellow	64
Salmon	372	Greenyellow	121	Royalblue	63
Pink	289	Darkgreen	116	Darkred	48
Yellow	292	Midnightblu	88	Darkgrey	38
Green	244	Lightcyan	77	Orange	31

### 3.3 Identifying Key Clinically Significant Modules

An eigengene adjacency heatmap (Figure 3A) was plotted by the plotEigengeneNetworks function (from the “WGCNA” R package) to explore the correlations between modules. In this research, the parameters of 94 samples included ischemic events, age, and time (postprocedure to ischemic event). The occurrence of ischemic events after CEA is an urgent problem to be solved, so our research focused on the early diagnosis of ischemic events. The yellow module (including 292 genes) (r = 0.26, *p* < 0.01) was the most notable module and had the strongest biological association with ischemic events in patients after CEA (Figure 3B).

### 3.4 GO and KEGG Enrichment Analysis and PPI Network Construction

**FIGURE 4:**
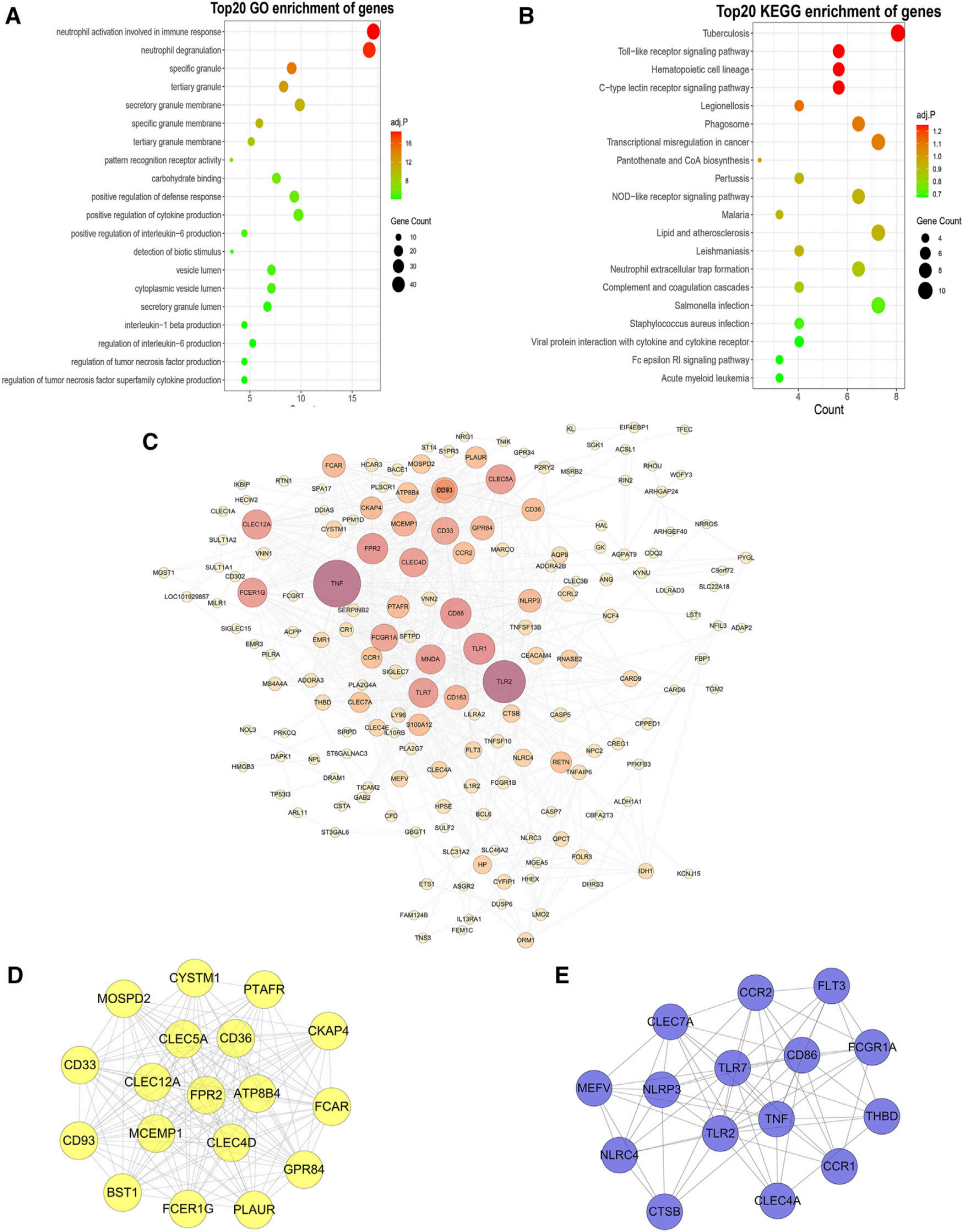
GO and KEGG enrichment analysis, PPI networks and hub genes. **(A,B)**: GO **(A)** and KEGG **(B)** enrichment analysis of yellow module genes. **(C)**: PPI network analysis of yellow module genes. The edge represents the interaction between two proteins. A greater node indicates that the protein is more important in the network. **(D,E)**: The two most closely connected network clusters in the PPI network identified using the MCODE plugin. [**(D)**: cluster 1: score = 16.353, node = 18, edges = 139; **(E)**: cluster 2: score = 9.429, node = 15, edges = 66].

To further investigate the functional features of the 292 genes in the yellow module, the enrichGO and enrichKEGG functions (from the “clusterProfiler” R package) were used to perform GO and KEGG enrichment analysis. Overall, the top 20 enriched GO terms and KEGG pathways from GO and KEGG enrichment analysis were plotted by the ggplot function (from the “ggplot2” R package) (Figures 4A,B). Among the GO terms, “neutrophil activation involved in immune response”, “neutrophil degranulation”, “specific granule”, “tertiary granule”, and “secretory granule membrane” were significantly enriched. Similarly, among the KEGG pathways, “Tuberculosis”, “Toll-like receptor signaling pathway”, “Hematopoietic cell lineage”, “C-type lectin receptor signaling pathway” and “Legionellosis” were significantly enriched. Based on the STRING database and Cytoscape software, a PPI network of the key genes within the yellow module was constructed (Figure 4C). Two key modules in the PPI network were identified by the MCODE plugin. The first module (score = 16.353, nodes = 18, edges = 139) consisted of 18 target genes, including *MOSPD2*, *CYSTM1*, *PTAFR*, *CKAP4*, *CD36*, *CLEC5A*, *CD33*, *CLEC12A*, *FPR2*, *ATP8B4*, *FCAR*, *CD93*, *MCEMP1*, *CLEC4D*, *GPR84*, *BST1*, *FCER1G*, and *PLAUR* (Figure 4D). The second module (score = 9.429, nodes = 15, edges = 66) consisted of 15 target genes, including *CLEC7A*, *CCR2*, *FLT3*, *MEFV*, *NLRP3*, *TLR7*, *CD86*, *FCGR1A*, *NLRC4*, *TLR2*, *TNF*, *THBD*, *CCR1*, *CLEC4A*, and *CTSB* (Figure 4E).

### 3.5 Identification of Optimal Diagnostic Biomarkers for Predicting Ischemic Events

**FIGURE 5:**
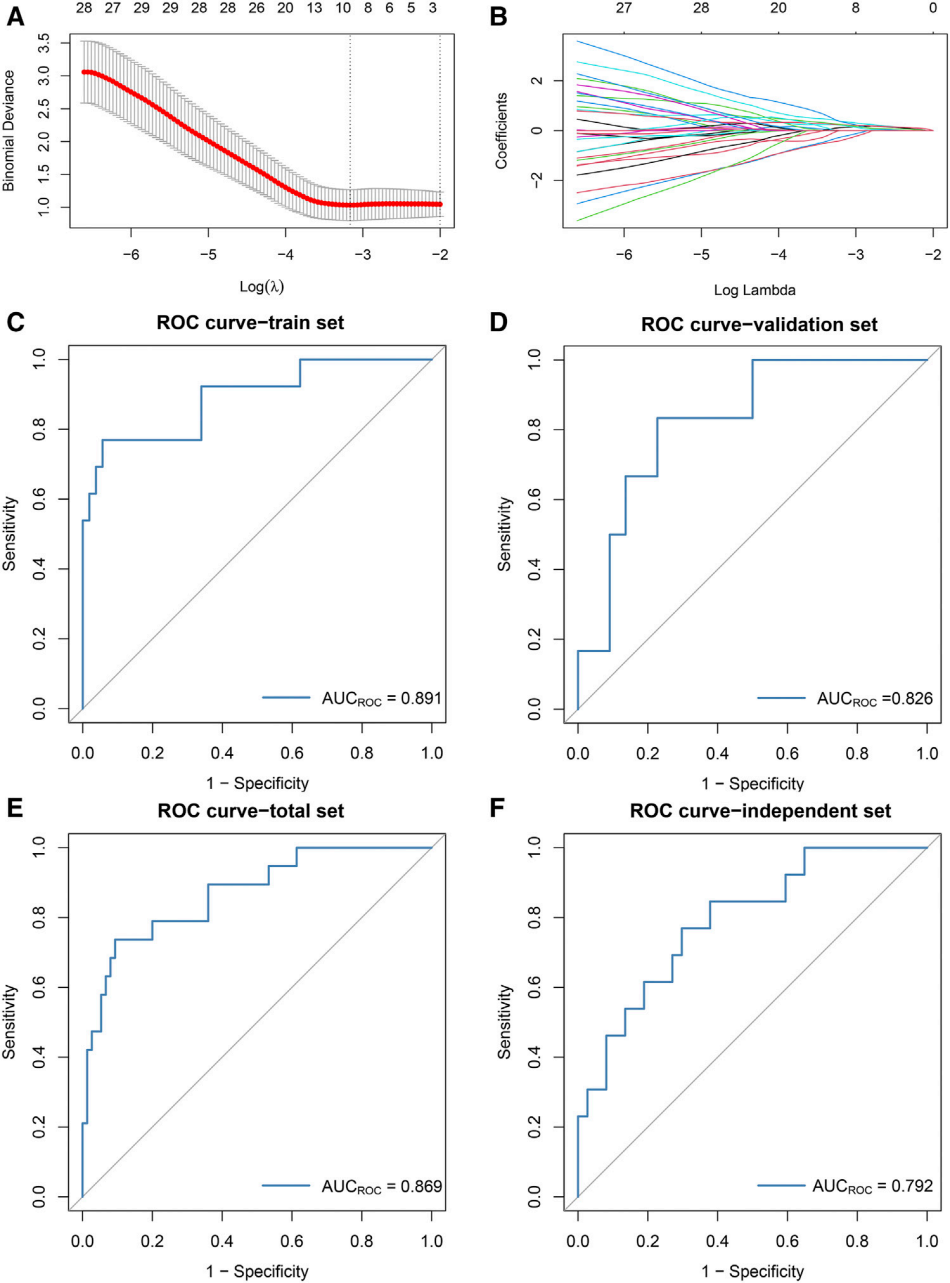
The development and validation of the diagnostic model based on the random forest and LASSO regression algorithms. **(A)**: The log(*λ*) value was optimally selected by 5-fold cross-validation and plotted by the partial likelihood deviance. **(B)**: The processes of LASSO regression for screening variables and mapping each variable to a curve. **(C–F)**: ROC curves were used to predict ischemic events after CEA in the train [**(C)**: AUC = 0.891], validation [**(D)**: AUC = 0.826], total [**(E)**: AUC = 0.869] sets and independent cohort [**(F)**: AUC = 0.792].

**FIGURE 6:**
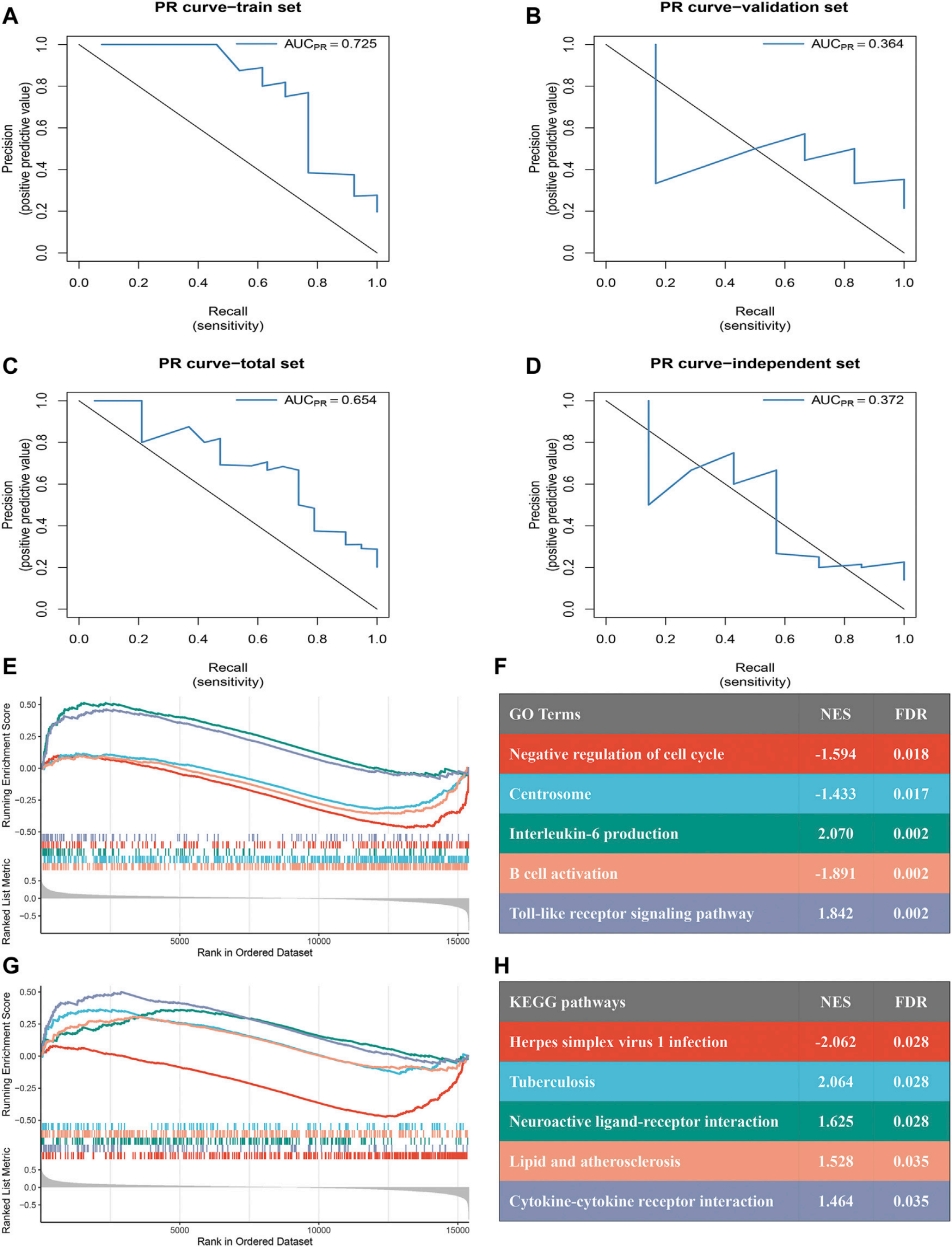
PR curves assess the accuracy of the eight-gene model and GSEA of the two subtypes. **(A–D)**: PR curves were used to predict ischemic events after CEA in the train [**(A)**: AUC = 0.725], validation [**(B)**: AUC = 0.364], total [**(C)**: AUC = 0.654] sets and independent cohort [**(D)**: AUC = 0.372]. **(E–F)**: Top five GO terms of differential genes in the high- and low-risk groups. **(G,H)**: Top five KEGG pathways of differentially expressed genes in the high- and low-risk groups.

According to the results of WGCNA, the yellow module is most associated with the occurrence of ischemic events after CEA. Using the expression of the genes in the yellow module in the train set, the random forest algorithm was applied. We retained 79 genes with relative importance >0.5. To further simplify the diagnostic model and reduce overfitting, LASSO regression was performed. Eventually, we obtained an eight-gene model, including *RLSCR1*, *ECRP*, *CASP5*, *SPTSSA*, *MSRB1*, *BCL6*, *FBP1* and *LST1* (Figures 5A,B). The final model formula was as follows: risk score = −1.61 - 0.24*PLSCR1 + 0.37*ECRP + 0.13*CASP5 + 0.20*SPTSSA - 0.38*MSRB1 + 0.34*BCL6 + 0.24*FBP1 + 0.23*LST1. According to this formula, we calculated the risk score of each patient. Logistic regression analysis showed that the eight-gene model was an independent predictor of ischemic events after CEA in the train dataset (odds ratio [OR] and 95% confidence interval [CI], 2.57 [1.33–7.24]; *p* = 0.005), validation dataset (OR and 95% CI, 10.64 [1.82–188.51]; *p* = 0.033) and total dataset (OR and 95% CI, 3.60 [1.60–9.08]; *p* = 0.003). ROC and PR curve analysis of the diagnostic model for predicting ischemic events was conducted in the train cohort, validation cohort, and total cohort. The ROC-AUCs value was 0.891 in the train cohort, 0.826 in the validation cohort and 0.869 in the total cohort (Figures 5C–E). The PR-AUCs value was 0.725 in the train cohort, 0.364 in the validation cohort and 0.654 in the total cohort (Figures 6A–C). These findings suggested that our model had a high accuracy performance.

### 3.6 Verification of the Eight-Gene Model Using RT-qPCR

RT-qPCR assays were performed in 50 samples. The risk score for the samples was calculated by the expression of the eight genes and risk score formula. ROC curve analysis of the diagnostic model for predicting ischemic events was conducted in the independent validation cohort. The ROC-AUC (Figure 5F) and PR-AUC (Figure 6D) value was 0.792 and 0.372 in the independent validation cohort.

### 3.7 GSEA

A total of 94 samples were divided into high- (*n* = 47) and low-risk (*n* = 47) groups according to the median risk score. GSEA revealed significant GO terms (Figures 6E,F) and KEGG pathways (Figures 6G,H) in which the differentially expressed genes were concentrated between the two risk subtypes. These were mainly inflammatory and immune infiltration-related functions or pathways, including “lipid and atherosclerosis” (normalized enrichment score (NES) = 1.528, FDR = 0.035), “cytokine-cytokine receptor interaction” (NES = 1.464, FDR = 0.035), “interleukin-6 production” (NES = 2.070, FDR = 0.002), “B cell activation” (NES = −1.891, FDR = 0.002) and “Toll-like receptor signaling pathway” (NES = 1.842, FDR = 0.002). These results indicated that our model has a close connection with inflammatory responses.

### 3.8 Immune Infiltration Analysis

**FIGURE 7:**
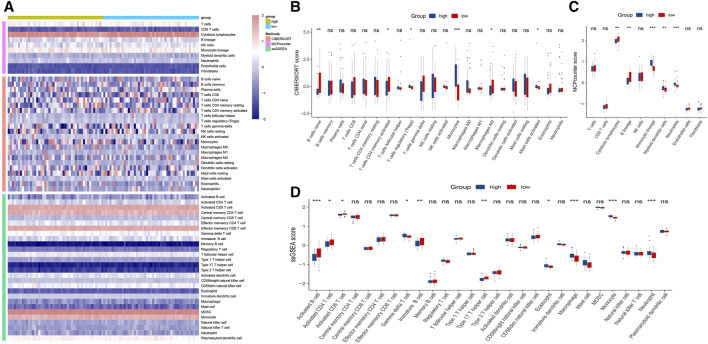
Immune infiltration analysis of the high- and low-risk groups. **(A)**: Heatmap for immune analysis based on CIBERSORT, MCP-counter and ssGSEA algorithms among two subtypes. **(B–D)**: Immune cell subset and related function association by the CIBERSORT, MCP-counter and ssGSEA algorithms. *p < 0.05, **p < 0.01, ***p < 0.001.

To explore the infiltration abundance of immune cells between the high- and low-risk groups, three algorithms, CIBERSORT, MCP-counter and ssGSEA, were performed to ensure the stability and reproduction of our results. We calculated the score of different cell subpopulations in 94 samples (Figure 7A). Interestingly, we found significant immune cell abundance differences between the two subtypes (Figures 7B–D), especially B cell subtypes (such as naive B cells, activated B cells, and immature B cells) and T cell subtypes (such as activated memory CD4 T cells, regulatory T cells, activated CD8 T cells, gamma delta T cells, and type 17 T helper cells). Overall, the high-risk group had higher immune assessment scores than the low-risk group.

## 4 Discussion

Ischemic events are treacherous events that occur in cardiovascular and cerebrovascular diseases, which are the leading causes of death and long-term disability worldwide (Collaborators, 2019; Campbell and Khatri, 2020; Iadecola et al., 2020). In recent years, substantial machine learning models have been applied to improve the clinical outcomes of diseases because they show better potential in diagnosis and prevention and improve the undesirable therapeutic status of patients (Vallee et al., 2019; Qiao et al., 2020). In addition, CEA is widely applied as a classic surgery to prevent ischemic events (Rerkasem et al., 2020). However, the detailed mechanisms underlying ischemic events and accurate diagnostic models for predicting ischemic events after CEA remain to be investigated.

In our study, we extracted a yellow module (including 292 genes) significantly related to ischemic events after CEA, according to the WGCNA results. GO and KEGG enrichment analyses were further used to identify the potential functions and mechanisms of these 292 genes. KEGG analysis showed that these genes mainly participated in “Tuberculosis”, “Toll-like receptor signaling pathway”, “Hematopoietic cell lineage”, “C-type lectin receptor signaling pathway” and “Legionellosis”. GO analysis further revealed that neutrophil activation, with terms such as “neutrophil activation involved in immune response”, “neutrophil degranulation”, “specific granule”, “tertiary granule” and “secretory granule membrane”, was the most significantly enriched functional module. A recent study found that patients with tuberculous meningitis (TBM) were more vulnerable to subsequent stroke (up to 57%), especially children or those with advanced stages and severe illness (Shulman and Cervantes-Arslanian, 2019). Zhang et al. reported that the inactivation of the Toll-like receptor signaling pathway protects neurological function in patients with ischemic events (Zhang et al., 2012). Moreover, both immune and inflammatory responses were activated in the acute and chronic phases following ischemic events, which played a double-edged role in pathophysiology (Pothineni et al., 2017; Jayaraj et al., 2019; Ketelhuth, 2019; Iadecola et al., 2020). Therefore, our results suggested that the genes in the yellow module played key roles in the progression of ischemic events.

Afterwards, to establish a diagnostic model for predicting recurrent ischemic events after CEA and further eliminate the effect of multicollinearity, we performed an integrated analysis of the relationships between gene expression and clinical characteristics in the cohort and used random forest and LASSO to screen the genes in the yellow module. Finally, we found that an eight-gene model (including *PLSCR1*, *ECRP*, *CASP5*, *SPTSSA*, *MSRB1*, *BCL6*, *FBP1* and *LST1*) was highly accurate for predicting ischemic events after CEA. Previous studies revealed that *BCL6* is a candidate gene for spontaneous hypertension and stroke (Watanabe et al., 2015), but further investigation into the mechanisms of these genes and ischemic events is necessary. Univariate logistic regression analysis revealed that the eight-gene model was an independent predictor. The higher the score calculated by the formula was, the higher the risk of ischemic events after CEA. More importantly, the ROC-AUCs and PR-AUCs of the train, validation, total, and independent cohort were 0.891 and 0.725, 0.826 and 0.364, 0.869 and 0.654, 0.792 and 0.372, respectively. The time window for the treatment of ischemic events is narrow, and it is difficult for most patients to receive treatment in a timely manner after onset, which leads to serious adverse consequences (Catanese et al., 2017; Gaafar et al., 2017). Therefore, it is particularly important to predict and accurately diagnose ischemic events after CEA.

Subsequently, we further explored the association of these eight genes with ischemic events after CEA. Previous study has shown that *PLSCR1*-*TRPC5* was a signaling complex mediating phosphatidylserine externalization and apoptosis in neurons and that plays a pathological role in cerebral-ischemia reperfusion injury (Guo et al., 2020). Zhang et al. (2020) reported that *CASP5* gene overexpression can significantly promote the angiogenesis ability of vascular endothelial cells by promoting the *VEGF* signaling pathway. This affected the formation of atherosclerosis and played a potential role in the development of ischemic events. Furthermore, *MSRB1* controlled immune response *in vivo* and anti-inflammatory cytokine release in macrophages (Guo et al., 2020). As we know, inflammatory factors were abundantly released and immune response was activated in ischemic events after CEA, thus, *MSRB1* may serve a protective role against events. *BCL6* may attenuate oxidative stress-induced neuronal damage by targeting the miR-31/*PKD1* axis and five novel single-nucleotide polymorphisms loci were identified in the *SLT1* locus to be associated with myocardial infarction (Iida et al., 2003; Wei et al., 2021). The above results further demonstrate that the eight-gene module affected ischemic events through multiple pathways, although three genes (*ECRP*, *SPTSSA* and *FBP1*) need to be further validated. Noteworthy, immune response played an important role in these pathways, which warrants further attention.

We further evaluated the immune infiltration among the two risk subtypes, which were divided by the diagnostic model, and more abundant immune infiltration was found in the high-risk group. A previous study demonstrated that a high abundance of immune infiltration is a risk factor for ischemic events (Iadecola et al., 2020). In the acute phase of ischemic events, immune cells attack the ischemic tissue, thereby aggravating the degree of ischemia. Metabolic substances released from ischemic tissue enter the circulatory system and eventually suppress the immune system, which leads to serious complications such as infection (Iadecola et al., 2020). These lines of evidence suggest that our research findings are persuasive. Therefore, the application of anti-immune and anti-inflammatory drugs may be a new strategy for the treatment of ischemic events after CEA.

Our work was a comprehensive study to develop an accurate eight-gene model for predicting ischemic events after CEA. Our research has the following advantages. 1) In this study, biomarkers were used to predict ischemic events after CEA, which was conducive to clinical transformation. 2) This diagnostic model has high accuracy, and the ROC-AUCs for the train, validation and total sets were all above or approach 0.8. 3) We validated the accuracy of the model in an independent cohort by RT-qPCR. 4) We found that the high-risk group of patients had abundant immune infiltration, which provided theoretical support for anti-immune and anti-inflammatory therapy in patients with ischemic events after CEA. However, although the diagnostic model was satisfactory in terms of its performance, several limitations remain in our research. First, some clinical features of samples were obscured in public datasets, which may affect our comprehensive exploration of the relationship between gene expression and clinical features (smoking, obesity, dyslipidemia, etc.). Second, compared the RNA-seq data, proteomics data can provide more favorable pathophysiological support, but proteomics analysis cannot be performed due to the lack of data. Although further studies are necessary, the proposed model still has great clinical value.

## 5 Conclusion

In conclusion, an efficient diagnostic model for predicting the occurrence of ischemic events after CEA was constructed. A population at high risk of recurrent ischemic events after CEA can be identified by this model. More importantly, the establishment of the eight-gene model provides new ideas for precise prevention and anti-immune and anti-inflammatory therapy in patients with ischemic events after CEA.

## Data Availability

The datasets presented in this study can be found in online repositories. The names of the repository/repositories and accession number(s) can be found in the article/Supplementary Material.
